# Potential bacterial biomarkers for insect colonization in forensic cases: preliminary quantitative data on *Wohlfahrtiimonas chitiniclastica* and *Ignatzschineria indica* dynamics

**DOI:** 10.1038/s41598-020-65471-6

**Published:** 2020-05-22

**Authors:** Lavinia Iancu, Georgiana Necula-Petrareanu, Cristina Purcarea

**Affiliations:** Institute of Biology Bucharest of Romanian Academy, Splaiul Independentei, 296, 060031 Bucharest, Romania

**Keywords:** Microbiology, Molecular biology

## Abstract

For the last decades, forensic microbiology became an emerging complementary tool in criminalistics. Although the insect-microbe interactions regarding pathogen transmission were extensively studied, only scarce information is available on bacterial transfer from necrophagous insects to host tissues. Our data provides the first report on the occurrence of *Wohlfahrtiimonas chitiniclastica* and *Ignatzschineria indica* in *Lucilia illustris* Meigen, 1826 (Diptera: Calliphoridae), and the quantitative dynamics of the two bacterial species along the insect life-stages and transfer to beef and pork host tissues using qPCR gyrase b specific primers. The content of both bacterial species increased along the insect life stages. *W. chitiniclastica* was detected in all developmental stages independent of the feeding substrate. *I. indica* was measurable with 10^2^ gene copies ng^−1^ DNA threshold starting from the third instar larvae when feeding on beef, and from the egg stage with a 10^2^× higher representation when using the pork substrate. The transfer of bacterial species to both tissues occurred after 3 colonization days except for *I. indica* that was visible in beef liver only during day 5. Considering the utilization of pork tissues as human analogues, these quantitative microbial dynamics data provides first insect-specific bacterial candidates as potential colonization biomarkers in forensic investigations.

## Introduction

The application of entomology in the field of forensic sciences related to the postmortem interval (PMI) estimation is well established since decades^1–4^, being based on the necrophagous insects presence and dynamics in decomposed human remains. Recently, studies involving the investigation of necrophagous insect’s microbiome emerged^5–9^, trying to elucidate the interaction of bacteria associated with different insect species. To date, the majority of the studies that have focused on investigating the insect microbiome mostly addressed problems like antimicrobial resistance and pathogen transmission^5,10–15^.

Very few studies have focused on the bacterial characterization and microbiome metagenomic assessment from different necrophagous insect species^6,7,16,17^, trying to add important data that can be applied in the field of forensic sciences. None, to our knowledge, targeted the specific amplification and quantification of *Wohlfahrtiimonas chitiniclastica* and *Ignatzschineria indica* transfer from *Lucilia illustris* Meigen, 1826 (Diptera: Calliphoridae) immature stages to the colonized tissues. These two species are gram-negative and aerobic bacteria, belonging to Proteobacteria phylum, Xanthomonadaceae family, being first isolated and described from necrophagous insect species. *W. chitiniclastica* was first identified and described from the flesh fly *Wohlfahrtia magnifica* (Schiner, 1862) (Diptera: Sarcophagidae)^18^, while *I. indica* was also first isolated from adult flesh flies^19^. These bacterial taxa were also reported from hospital cases involving sepsis^20^, bacteraemia^21^, myiasis^22^, their identification and investigation having medical importance in certain clinical cases.

Sometimes, in unforeseen circumstances^23^ or during autopsies, the human bodies are either clean of insect eggs, larvae and/or adults, or those go unnoticed. Information on the transfer of insect-specific bacteria could be very useful in proving a previous presence of the necrophagous insect species on the decomposed body, even from the earliest moment of colonization. This can be accomplished by quantitatively analyzing the bacterial content, which, as it appears from the investigation carried out in this article, changes according to the insect developmental stage and/or tissue diet. By relating the bacterial quantity variation to the different insect developmental stages, and by correlating these variations with the colonization time, bacterial species insect-associated could be used in the near future as PMI biomarkers.

In this respect, the main objective of the present research consisted in the quantitative investigation of two insect-specific bacterial taxa throughout *L. illustris* immature life stages (egg, first instar larvae, second instar larvae, third instar larvae) and in the quantitative analysis of the bacterial transfer to the feeding substrate in function of the different larval stages. This article provides first insights into the bacterial transfer quantification having potential implications in the field of forensic sciences. The importance of the present study also resides in the fact that these bacterial taxa were identified from different necrophagous insect species, sampled from different geographical locations^6,7,9,16,24–26^, so that the data obtained during this survey can be of use to the international scientific community, due to their role as possible PMI biomarkers in the absence of insects. Nevertheless, before any conclusion is drawn, numerous studies must be carried out, using even other insect-specific bacteria, and even other insect species that colonize decomposed remains.

## Material and Methods

### Experimental design

*Lucilia illustris* Meigen, 1826 (Diptera: Calliphoridae) was selected for the survey considering its synanthropic environmental occurrence and high abundance during summer months, being also an earliest primary carrion colonizer. *L. illustris* adults were sampled three different times (approx. 30 specimens/sampling time) from the urban area of Bucharest (Romania) (N44°26′49.349′ E26°2′45.352″) during summer (July 2017).

**Figure 1:**
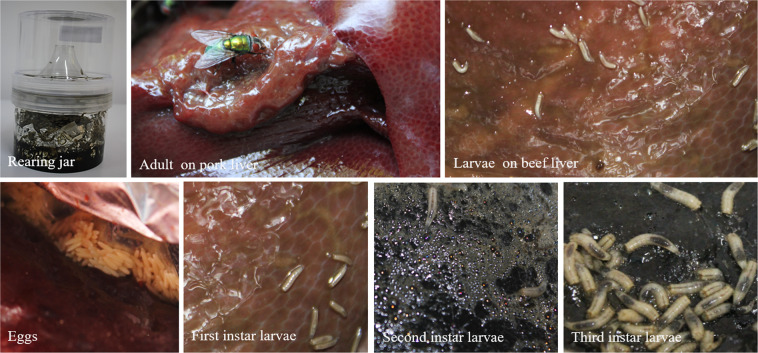
Experimental setup for *Lucilia illustris* immature stages reared on beef and pork liver.

The adult specimens collected with entomological nets were placed in sterilized rearing chambers in the presence of beef or pork liver tissues, under constant laboratory conditions (25 °C ± 1 and 60% relative humidity, LD 12:12). After oviposition, *L. illustris* eggs were placed in small sterilized rearing jars of 21 × 12 cm in diameter (BioQuip Products, Rancho Dominguez, CA, USA) and the immature stages were reared under the same constant laboratory conditions, in the presence of 375 g of liver, being sampled daily. Non-colonized rearing jars containing the same amount of pork or beef liver were used as control and kept under the same experimental aseptic conditions (Fig. 1).

### Insect and tissue sampling

Insect and liver tissue were collected daily in triplicate along 2-weeks period, considering all *L. illustris* immature developmental stages (egg, first instar, second instar and third instar larvae) and fresh and aged liver samples. Approximately 0.3 g of eggs and one individual larvae of each stage were sampled for DNA extraction. Liver tissue samples were collected prior to insect colonization and simultaneously with the daily insect sampling.

Liver tissue and insect samples were preserved in 200 µl Tris-EDTA pH 8 (TE) buffer at −20 °C for DNA extraction, and in 75% ethanol for taxonomic identification.

Insect adults and immature stages were identified using the taxonomic identification keys provided by Lehrer^27^ under a stereomicroscope Stemi2000 (Zeiss, Germany), and confirmed by COI barcoding.

### Quantitative polymerase chain reaction (qPCR) of bacterial gyrase b gene fragments

Quantification of *Wohlfahrtiimonas chitiniclastica* and *Ignatzschineria indica* in insects and liver tissues was carried out by qPCR specific bacterial gyrase subunit B gene (*gyr*B) fragments. The primers (Table 1) were designed and the melting temperatures were calculated using OligoCalc software^28^. Total DNA was extracted from the liver and insect samples according to DNeasy Blood & Tissue (Qiagen, Valencia, CA, USA) modified protocol^29^. DNA concentration and purity were measured with a BiodropDuo UV/VIS Spectrophotometer (Harvard Bioscience Inc, Holliston, MA, USA). For insects, the specimens were washed in 70% ethanol and sterile 10 mM phosphate-buffered saline (PBS) solution to remove all exterior contaminants, and homogenized for 12 min in the presence of 2 mm ZR Bashing Beads (Zymo research, Irvine, CA, USA) using a SpeedMill PLUS Cell Homogenizer (Analitik Jena, Jena, Germany) prior to total DNA extraction.

**Table 1:** *W. chitiniclastica* and *I. indica gyr*B gene-based primers for qPCR amplification.

Bacterial species	Primers	Sequence
*W. chitiniclastica*	F_Who_chit	5′-CCTTCTTAAACTCTGGCATTCGCA-3′
	R_Who_chit	5′-GAAAACAGATGGATTGATGGGGGTT-3′
*I. indica*	F_ Ign_ind	5′-AGCTGCTAGAAAAGCGAGAGAAAAC-3′
	R_ Ign_ind	5′-TAATGTACCAATCTCAGCGGAGCTA-3′

The reaction mixtures contained Thermo Scientific Maxima SYBR Green (1×) Master Mix (ThermoFisher Scientific), 10 µM of each forward and reverse primer, 100 ng DNA template and nuclease free water in a total volume of 10 µl. The amplification reactions comprising an initial incubation step of 10 min at 95 °C followed by 40 cycles of 15 sec at 95 °C, 30 sec at 49 °C for *I. indica* or 50 °C for *W. chitiniclastica*, and 30 sec at 72 °C, were carried out using a Mastercycler ep gradient S thermocycler PCR (Eppendorf, Wien, Austria). The melting curve analysis was used as amplification control, being included at the end of each program. Quantification was performed by interpolation in a standard regression curve of cycle threshold (Ct) values generated from samples of known concentration of DNA template. Standard curves (Supplementary Fig. S1) were generated using corresponding PCR amplified DNA fragments after extraction and purification from 1% agarose gel using QIAquick gel extraction kit (Qiagen).

The DNA decimal dilutions series for *W. chitiniclastica* ranged between 7.5 × 10^−4^ ng/μL–7.5 × 10^−9^ ng/ μL, and 3.7 × 10^−2^–10^−8^ ng/μL for *I. indica* (R^2^ > 0.99). No-template controls were included in all PCR runs. Samples were run in triplicates, with a Ct threshold value over 34 not considered.

The gene copy number per reaction was calculated as^30^:

$${\rm{Copy}}\,{\rm{number}}=({\rm{ng}}\,{\rm{DNA}}\ast 6.023\times {10}^{23})/({\rm{length}}\ast 1\times {10}^{9}\ast 650),$$

where the length represents the base-pair length of the PCR product, and the plot was performed between the logarithmic scale (base 10) of copy gene number and the experimental time.

## Results

### Bacterial specific *gyr*B primers

**Figure 2:**
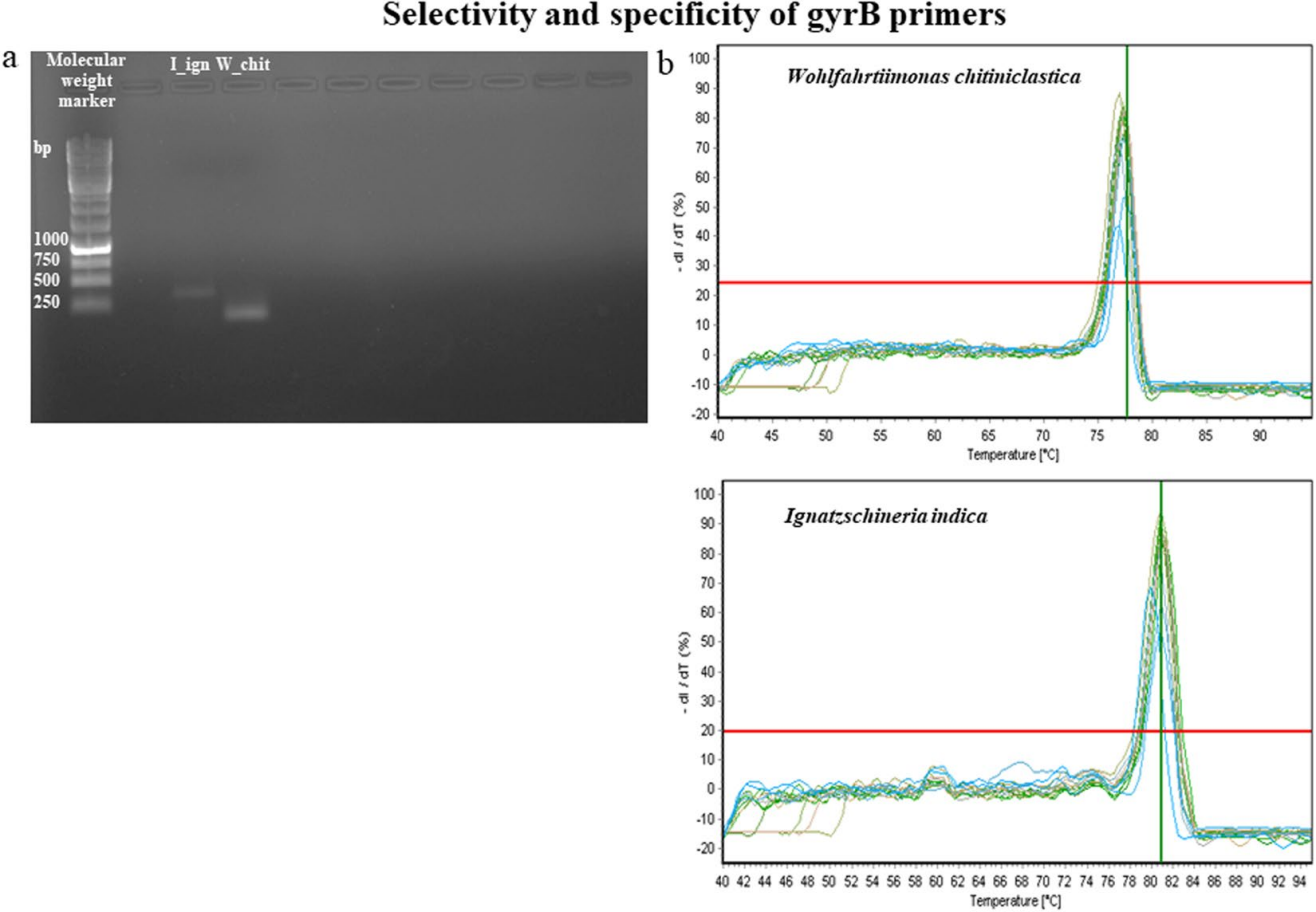
PCR amplification of *Wohlfahrtiimonas chitiniclastica* and *Ignatzschineria indica* *gyr*B gene fragments. (**a**) bacterial *gyr*B amplicons and **(b**) melting temperatures of the amplified *gyr*B gene fragments of the two bacterial species.

Given the high similarity of the 16S rRNA genes of the investigated species (82.6% similarity), specific primer pairs for each bacterial species (Table 1) were designed using the DNA gyrase subunit B gene (*gyr*B) fragments. The corresponding amplicon length covered 140 bp for *W. chitiniclastica* and 262 bp for *I. indica*. Both PCR amplification (Fig. 2a) and melting temperature curves (Fig. 2b) indicated high specificity for amplification of *W. chitiniclastica* and *I. indica* corresponding genes.

### *Wohlfahrtiimonas chitiniclastica* in insect immature stages

**Figure 3:**
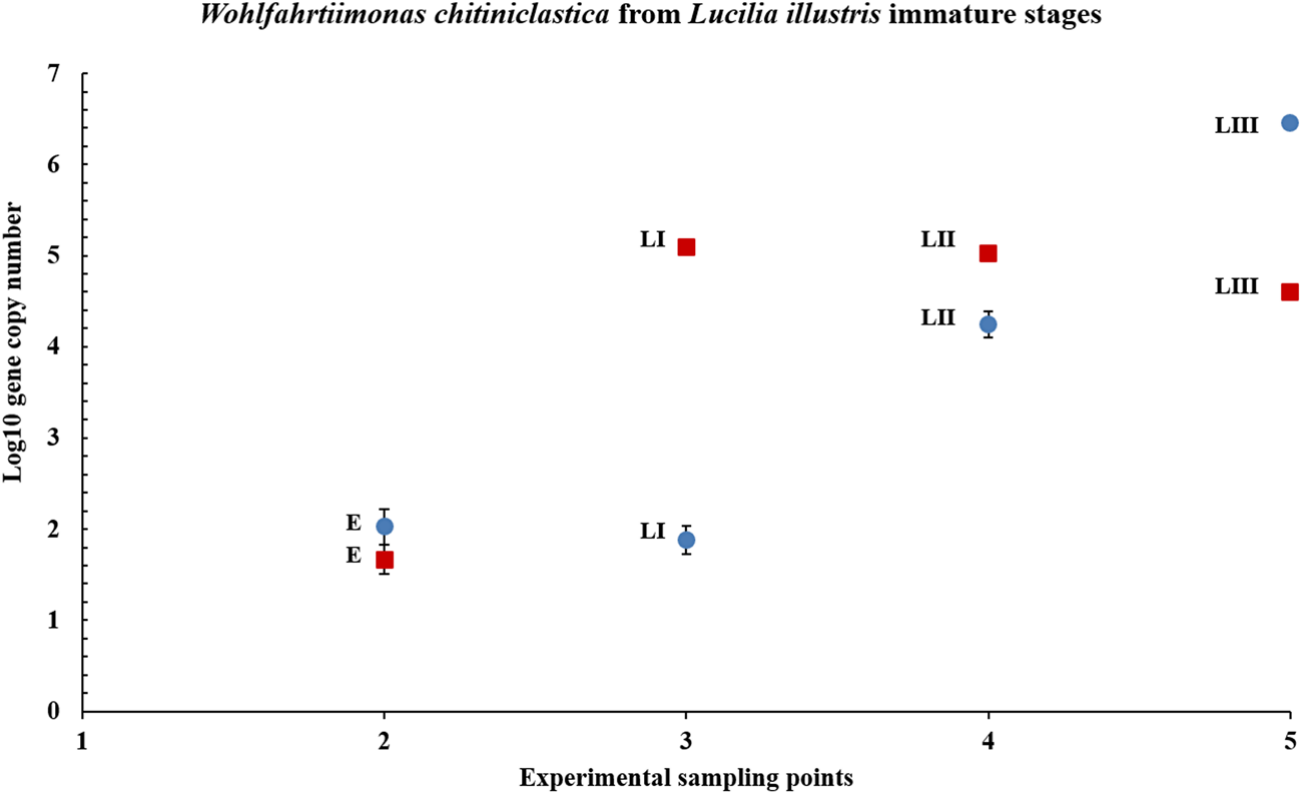
Relative abundance of *Wohlfahrtiimonas chitiniclastica* quantified from *Lucilia illustris* immature stages (E – egg; LI – first instar larvae; LII – second instar larvae; LIII – third instar larvae) reared on pork and beef liver. Standard deviations are indicated by error bars.

*W. chitiniclastica* was present in all *L. illustris* developmental stages (Fig. 3), starting with the earliest immature stage (i.e. egg). Lower abundances were recorded for the egg samples (10^1.68^) collected from the pork liver feeding substrate, with a maximum bacterial content reached starting with the first instar larvae, about 10^3^x higher than in the egg samples. At the same time, *W. chitiniclastica* exhibited comparable amounts between the three larval stages (10^5.09^, 10^5.02^, 10^4.6^).

For the beef liver substrate, a lower bacterial transfer was recorded for the first and the second instar larvae, with comparable abundances for the egg and first instar larvae samples (10^2^). Further, the maximum abundances for *W. chitiniclastica* were recorded for the second and third larval stages (10^4.2^–10^6.4^).

Compared to the immature stages that had a pork liver diet and presented a rather consistent trend after the initial *W. chitiniclastica* quantitative growth in the first instar larvae, the immature stages that were fed with beef presented an upward bacterial dynamic trend, even if smaller quantities were recorded for the first two instars. These quantitative differences observed for *W. chitiniclastica* from pork and beef reared specimens may be a consequence of the feeding substrate.

### *Ignatzschineria indica* in insect immature stages

**Figure 4:**
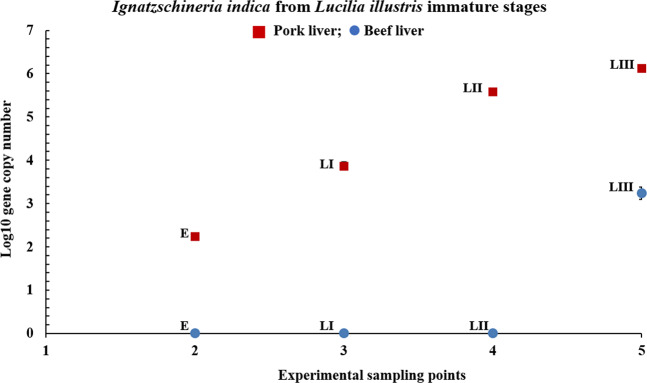
Relative abundance of *Ignatzschineria indica* quantified from *Lucilia illustris* immature stages (E – egg; LI – first instar larvae; LII – second instar larvae; LIII – third instar larvae) reared on pork and beef liver. Standard deviations are indicated by error bars.

In the case of *I. indica* (Fig. 4) the bacterial cell density showed again a possible dependence on the tissue type used as feeding substrate. For the pork tissue substrate, *I. indica* was recorded in all *L. illustris* immature stages, with very low abundances for the egg samples (10^2.2^), and maximum content recorded for the third instar larvae (10^6.1^), showing a 10^2.72^ × increase as compared to the egg samples, and also an upward trend throughout larval development.

On the other hand, *I. indica* identified from the insect immature stages reared on beef liver was visible only in the third instar larvae (10^3.2^), exhibiting 10^−1.89^× lower quantities compared to the third larval stages fed with pork liver diet. During the earlier insect life stages, *I. indica* was not observed given the very low abundances, overall being present in 50% lower abundance than in the insect specimens reared on the pork liver.

### Pork and beef liver tissues

**Figure 5:**
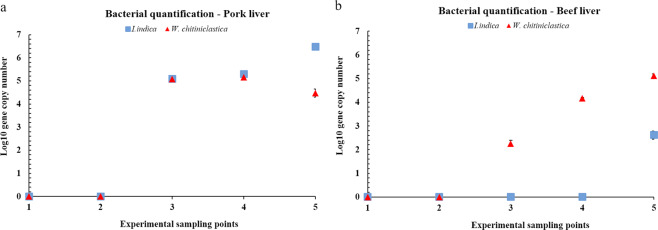
Relative abundance of *Ignatzschineria indica* and *Wohlfahrtiimonas chitiniclastica* transferred to (**a**) pork liver and (**b**) beef liver. Standard deviations are indicated by error bars.

In the case of the pork liver substrate (Fig. 5a), both *W. chitiniclastica* and *I. indica* transfer occurred after three experimental days, with a similar dynamics trend during days 3 and 4 (10^5.1^–10^5.3^), 10^2.47^x higher for *I. indica* than *W. chitiniclastica* in day 5. The higher bacterial content registered from the pork liver belonged to *I. indica* in day 5 (10^6.4^).

*W. chitiniclastica* was present in the beef liver (Fig. 5b) starting with day 3, showing a constant increase up to day 5 (10^2.2^–10^5.1^), reaching comparable levels as in the pork liver.

No identification of *I. indica* was accounted for the beef feeding substrate during the first 4 experimental days, in accord with the first developmental stages of *L. illustris* specimens (egg, I and II instar larvae). *I. indica* was observed only during day 5 (10^2.6^), presenting 10^−1.95^ × lower content than *W. chitiniclastica*.

Although the abundance profile varied between pork and beef feeding substrates, the highest bacterial content for the two taxa investigated was reached for the insect specimens reared on pork liver. Moreover, the initial content of both bacterial taxa could not be detected in the first experimental days in the liver samples, nor from the liver used as reference.

## Discussions

The decomposition dynamics of human and animal carcasses is dependent on the cause of death, ante mortem circumstances, geographical location, meteorological parameters as well as specific conditions encountered at the decomposition environmental site^9,31,32^. For the most part, the decomposition begins immediately after the negative signs of life are installed, manifested by the biochemical changes and the proliferation of bacteria from the gastrointestinal tract. All of these physical and biochemical changes attract a diversity of necrophagous insect species, which come to colonize the respective cadaver in a certain time sequence. When the temperatures are higher, a decomposed body attracts faster Diptera and/or Coleoptera species that reproduce and feed on the remains^33^. Starting with the initial moment of carcass colonization by different necrophagous insect species (especially blowflies), a microbiome transfer from the insect species to the colonized remains also takes place^9,34^.

For the insect species the periods of activity, colonization patterns and feeding preferences have been well researched^35–39^. However, more is to be known regarding the transfer of insect-specific bacterial species throughout the developmental cycle, as well as their transfer to the colonized tissues/substrates. To date, those studies that focused on characterizing the bacterial diversity from different insect species used both classical microbiological cultivation^17,24,25,40^ or/and modern molecular high throughput 16S rRNA gene sequencing^6,7,24,25,41–43^, as investigation techniques.

Given their medical, veterinary and forensic importance, muscids^24^ and calliphorids^7^ were used to investigate the insect microbiome content, focusing primarily on the pathogen transmission^17^ and antimicrobial resistance^44^. Moreover, studies concerning the bacterial communities involved their relationships and role played during the developmental stages of different insect species^40^.

In this regard, *Musca domestica* Linnaeus, 1758 (Diptera: Muscidae) was often used as experimental model for these types of studies given this species frequently association with human living environments, easily transmitting different pathogens. As such, the research focused mainly on characterizing the bacterial diversity and on the identification of bacterial pathogens^40^ from this muscid species.

However, other insect species, such as *Wohlfahrtia magnifica* (Schiner, 1862) (Diptera: Sarcophagidae) were used to investigate the bacterial content. Tóth and collaborators^17^ studied the bacteria associated with *W. magnifica* given the myiasis-causing potential of this fly species in domestic animal, especially in Eurasia. During their study it was emphasized that *Ignatzschineria* spp. is most probably associated with the insect larvae foregut. At the same time, both *Ignatzschineria* and *Wohlfahrtiimonas* were noticed to have a strong chitinase activity, that may play an important role during the fly developmental period^17^. Even though Tóth and collaborators^17^ did not performed a quantitative investigation, both bacterial species were identified from *W. magnifica* larval stages. In our experiment not only that the bacterial species were identified, but their quantitative presence was analyzed, demonstrating that these taxa are insect specific and that their in-depth study can lead to a better understanding of their involvement as insect colonization biomarkers.

Furthermore, the bacterial diversity was also investigated from the black soldier fly *Hermetia illucens* (Linnaeus, 1758) (Diptera: Stratiomyidae) life stages^6^. The main purpose of Zheng and collaborators^6^ study was to analyze the transmission of pathogens throughout *H. illucens* developmental cycle. Nevertheless, the bacterial diversity characterization revealed *Ignatzschineria* spp. presence in the analyzed specimens. This identification proves once again this bacterial association with fly species belonging to different Diptera families.

Weatherbee and collaborators^45^ researched the larvae mass microbiome belonging to different Calliphoridae species (*Phormia regina* Meigen, 1826, *Lucilia coeruleiviridis* Macquart, 1855, *Cochliomyia macellaria* (Fabricius, 1775)) and revealed Xanthomonadaceae as an abundant family, though no taxonomic level up to genera was presented. Nevertheless, their study showed that Xanthomonadaceae increased throughout the decomposition process. These results are similar to our data on the investigated and quantified bacterial taxa that exhibited a similar increasing time pattern both in the insect immature stages and colonized liver.

A more recent study^42^ focused on studying the bacterial diversity associated with the third instar larvae of the stable fly *Stomoxys calcitrans* (Linnaeus, 1758) (Diptera: Muscidae). The results showed *Ignatzschineria* among the most abundant bacterial genera in the larvae specimens. At the same time, *Ignatzschineria* was more abundant in the fly larvae specimens than in the living substrates, similar to our findings, were *I. indica* prevailed in *L. illustris* immature stages. Other synanthropic species, such as the oriental latrine fly, *Chrysomya megacephala* (Fabricius, 1794) was used for the microbiome characterization during the developmental cycle^43^. During the respective study^43^, both *Ignatzschineria* and *Wohlfahrtiimonas* were identified from the insect samples, though, unlike other experiments, these genera were not encountered among the prevailing gut bacteria. Nevertheless, once again it is demonstrated that these bacteria are present in different Diptera species, which may recommend them as universal biomarkers for the insect colonization time.

During another study that investigated the persistence of antibiotic resistant bacteria performed by Wei and collaborators^46^, using *Proteus mirabilis* in *M. domestica* and the green fly *Lucilia sericata* (Meigen, 1826) (Diptera: Calliphoridae) the presence of *Ignatzschineria* and *Wohlfahrtiimonas* was revealed. They used qPCR assays to quantitatively monitor *P. mirabilis* and to evaluate for how long different type strains persist in the fly’s digestive tract, but also used 454 pyrosequencing assays to characterize the entire microbiome. Similar to our study, the authors used qPCR as a method of investigation, though targeting a different bacterial taxon. However, the microbiome characterization by 454 pyrosequencing revealed *Wohlfahrtiimonas* in *L. sericata* specimens. Furthermore, *Ignatzschineria* genus was identified as prevalent bacteria in green bottleflies^46^ sampled during summer months, consistent to our findings of *Ignatzschineria indica* from *L. illustris*.

*W. chitiniclastica* was identified as a common bacterial taxon from both culture isolates and clone library samples during Gupta and collaborators study^24^, *Ignatzschineria* spp. being also identified during the survey. Both *Ignatzschineria* and *Wohlfahrtiimonas* were identified during another Gupta and collaborators^25^ investigation on bacterial content from adult sarcophagids intestinal gut. Moreover, both taxa were among the prevalent cultured bacteria identified from both flesh flies’ adults and larvae samples, although *Ignatzschineria* was common in both culture and clone sequences.

Even though most of the studies performed to date concerned mainly bacteria that are muscids-associated, green flies were also considered for research. In this respect, Singh and collaborators^7^ investigated the bacterial content from *Lucilia sericata* (Meigen, 1826) and *Lucilia cuprina* (Wiedemann, 1830) species (Diptera: Calliphoridae). During their study the horizontal transmission of bacteria was more noticeable than the trans-generational inheritance. Although during their experiment the taxonomic identification was performed up to the genera level, *Ignatzschineria* was encountered among the first five dominant genera identified from the two insect species, while no records of *Wohlfahrtiimonas* were mentioned. Moreover, as the authors stated, data on bacteria associated with *Lucilia* species is limited, and though several years had passed since their study, scarce in-depth investigations were directed towards the research of bacteria associated with green bottleflies. In this regard, the current study presents innovative and preliminary data on *W. chitiniclastica* and *I. indica* that may help to expand the research in the forensic field, regarding the necrophagous insect-associated bacterial investigations.

All these previous studies revealed the presence and frequent association of *Ignatzschineria* and *Wohlfahrtiimonas* with different insect species. The presence and quantification of both bacterial genera in necrophagous insect species can provide data on body colonization even in the absence of adults and/or immature stages. This could happen, as previously mentioned, during the crime scene investigation or autopsy sampling where, regardless of the reason, the insect evidence is overlooked, especially at the beginning of insect colonization (i.e. egg clusters). However, this information could be used in the absence and/or presence of entomological evidence, in order to bring additional information about the colonization time, throughout the bacterial quantitative time related presence.

In the current survey, both *Ignatzschineria* and *Wohlfahrtiimonas* taxa were identified from insects and liver tissue samples, with comparable values for the pork liver substrate, suggesting that they can be used as potential bacterial markers for insect colonization, supporting the base hypothesis of this experiment.

Although further investigations targeting these bacterial species are required to confirm their role as colonization biomarkers using various feeding substrates, larvae tissues and conditions, this report highlights for the first time the applicative potential in forensic sciences of these two bacterial species.

## Supplementary information

Supplementary Figure S1.
