# Supplementary material for: Potential bacterial biomarkers for insect colonization in forensic cases: preliminary quantitative data on Wohlfahrtiimonas chitiniclastica and Ignatzschineria indica dynamics
Source: Sci Rep. 2020 May 22;10:8497. doi: 10.1038/s41598-020-65471-6 (PMC7244533; doi:10.1038/s41598-020-65471-6)
Supplement: Supplementary file 1 — Supplementary Figure S1. [file 41598_2020_65471_MOESM1_ESM.pdf]

Potential bacterial biomarkers for insect colonization in forensic cases: preliminary quantitative data on *Wohlfahrtiimonas chitiniclastica* and *Ignatzschineria indica* dynamics

Lavinia Iancu*, Georgiana Necula-Petrareanu, Cristina Purcarea

a

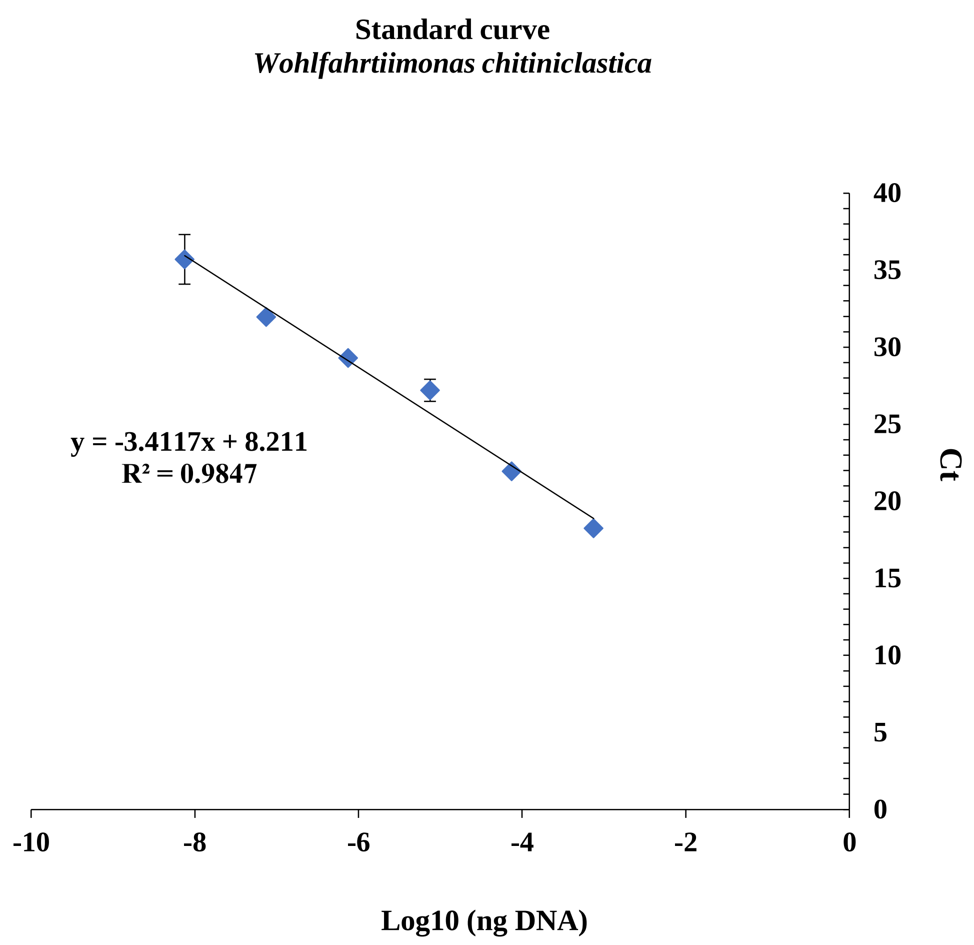

b

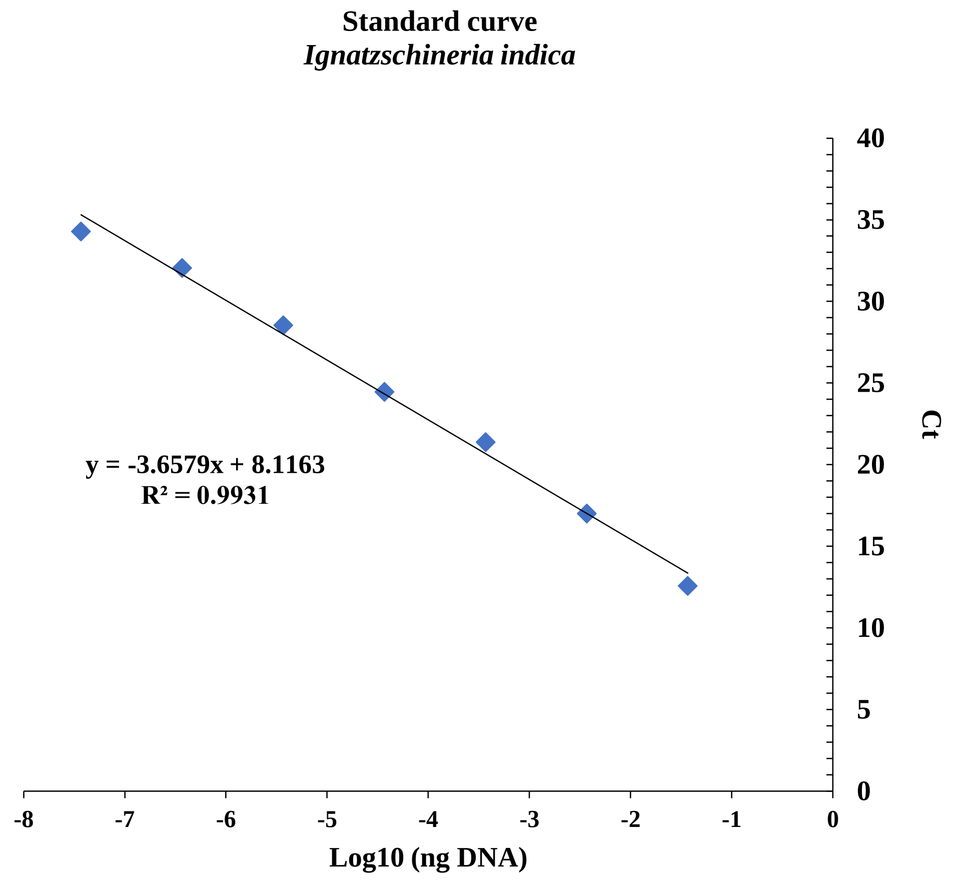

Supplementary Fig.1. Standard curve for (a) *Wohlfahrtiimonas chitiniclastica* and (b) *Ignatzschineria indica*.
